# Th17-Gene Expression Profile in Patients with Chronic Venous Disease and Venous Ulcers: Genetic Modulations and Preliminary Clinical Evidence

**DOI:** 10.3390/biom12070902

**Published:** 2022-06-28

**Authors:** Rosario Amato, Vincenzo Dattilo, Carolina Brescia, Lucia D’Antona, Rodolfo Iuliano, Francesco Trapasso, Nicola Perrotti, Davide Costa, Nicola Ielapi, Francesco Aiello, Michele Provenzano, Umberto Marcello Bracale, Michele Andreucci, Raffaele Serra

**Affiliations:** 1 Department of Health Sciences, University “Magna Graecia” of Catanzaro, 88100 Catanzaro, Italy; rosario.amato@unicz.it (R.A.); dantona@unicz.it (L.D.); iuliano@unicz.it (R.I.); perrotti@unicz.it (N.P.); 2 Medical Genetics Unit, University Hospital “Mater Domini” of Catanzaro, 88100 Catanzaro, Italy; dattilo@unicz.it (V.D.); trapasso@unicz.it (F.T.); 3 Department of Experimental and Clinical Medicine, University “Magna Graecia” of Catanzaro, 88100 Catanzaro, Italy; brescia@unicz.it; 4 Department of Law, Economics and Social Science, University “Magna Graecia” of Catanzaro, 88100 Catanzaro, Italy; davide.costa@studenti.unicz.it; 5 Interuniversity Center of Phlebolymphology (CIFL), International Research and Educational Program in Clinical and Experimental Biotechnology, Department of Surgical, Medical Sciences, University Magna Graecia of Catanzaro, 88100 Catanzaro, Italy; nicola.ielapi@uniroma1.it; 6 Department of Public Health and Infectious Disease, “Sapienza” University of Rome, 00185 Roma, Italy; 7 Department of Medical and Surgical Sciences, University “Magna Graecia” of Catanzaro, 88100 Catanzaro, Italy; francesco.aiello6@studenti.unicz.it (F.A.); michiprov@hotmail.it (M.P.); 8 Department of Public Health, University of Naples “Federico II”, 80138 Naples, Italy; umbertomarcello.bracale@unina.it; 9 Department of Health Sciences, University of Catanzaro, 88100 Catanzaro, Italy; andreucci@unicz.it

**Keywords:** chronic venous insufficiency, chronic venous leg ulcers, Th17, SGK1, IL17, IL23R

## Abstract

Chronic venous disease is a condition globally widespread, resulting in a disabling pathological disorder. The CD4 + Th17+ (Cluster Differentiation 4) lymphocytes represent a regulative factor for innate immunity related to the development of complex diseases. Recently, these mechanisms have been associated with vascular disease. The aim of this work is to validate whether the Th17 response correlates with the development of CVI (Chronic venous insufficiency)and CVLUs (chronic venous limbs ulcers) and whether Th17 markers can be used, both as intrinsic risk factors and diagnostic markers, for disease development. PBL derived from peripheral blood samples of patients and controls were subjected to gene expression analysis for IL23R, IL17, SGK1, TGFβ, RORγ, FOXO1, and RANBP1 by qRT-PCR and immunoblot. A post hoc correlation, the diagnostic performance of the target genes, and multivariable analyses were properly conducted. The main expression markers of the CD4 + Th17+ switch were strongly activated in chronic venous insufficiency and in advanced ulceration. The correlation analysis demonstrated the inter-dependence on Th17’s signature modulation. ROC (Receiver Operating Characteristic) analysis defined, for the examined genes, a clinical value as the potential diagnostic markers. Multi-logistic regression studies showed that Th17 markers behave as empirical risk factors for CVD (chronic venous disease) development. Taken together, the present data provide a new hypothesis for the TH17-dependent pathogenesis of CVD, favoring the possibility for the development of new diagnostic, preventive, and therapeutic approaches.

## 1. Introduction

Chronic venous disease (hereafter always referred to as CVD, unless otherwise defined) is a widespread clinical condition, with a prevalence ranging from 10% in adults younger than 30 years of age to nearly 80% for individuals > 70 years of age [1]. CVD clinical manifestations vary from mild signs, including varicose veins, to more advanced and severe signs, such as chronic venous leg ulcers (CVLUs), which significantly impact the patient’s quality of life (QoL) [2,3]. Several genetic alterations have been studied to understand the onset, progression, and complications of CVD, including chronic venous insufficiency (CVI) states and CVLUs [3,4,5]. The appearance of CVLUs is generally preceded by skin changes in the lower limbs, such as lipodermatosclerosis [3]. This pathological event leads to subcutaneous fibrosis and the hardening of the affected skin, resulting in tissue hypoxia essential for venous ulceration [6,7]. Gene expression profile studies, described in the current literature, allow us to hypothesize several mechanisms underlying the development of CVLU, highlighting a wide variety of genetic-molecular interconnections [4]. Nevertheless, none of them are able to provide a complete genetic and cellular model linking the pathogenetic events during CVLU’s progression [6]. T-helper-17 (Th17) cells are a subtype of pro-inflammatory T helper (CD4+) cells, defined by the production of a cytokine signature, of which IL17 represents the progenitor [8]. The development and expansion of Th17 depend on differentiation factors (TGF-β), growth factors (IL23/IL23R), and several transcription factors (ROR-γt, STAT3) [9].

The Th17-axis has been implicated in several autoimmune diseases, including rheumatoid arthritis, multiple sclerosis, ulcerative colitis, Crohn’s disease, and autoimmune encephalitis, among others [10,11]. In addition, an increasingly strong role of the Th17 axis in tumor drug resistance and in the progression of HIV infection is emerging [12,13]. Moreover, the Th17 switch plays a key role in several acute and chronic inflammatory diseases, including the COVID-19 cytokine storm [11,12,14,15,16]. A high salt concentration causes a Th17 switch, resulting in increased IL17A production and, consequently, Treg cells’ down-regulation. This dose-dependent relationship is found to be amplified in the presence of Th17-specific factors, including TGF-β, IL6, IL21, and IL23/IL23R. Microarray analysis corroborated that, at high salt concentrations, cells express a strong Th17 phenotype with increases in IL17A, RORG, and IL23 [17]. From a molecular perspective, hypertonicity of the medium, induced by a high salt concentration, induces the phosphorylation of p38/MAPK and downstream targets, especially NFAT5, which, in turn, induces SGK1. SGK1 enhances the IL23R mRNA expression and promotes the transcription of ROR-γt, an essential regulator of Th17 differentiation [17,18,19]. Conversely, the inhibition of p38/MAPK and NFAT5 induces the downregulation of SGK1’s activation and IL17 production [18,19]. Recently, it has been demonstrated that SGK1 plays a switch role in the phasic modulation of the Treg/Th17 expression, also through regulating FOXO1 compartmentalization [17]. The previous data have shown that in ulcerative colitis, SGK1 plays a dual role in the disease’s pathogenesis, both inducing Th17 differentiation in circulating/tissue lymphocytes and modulating organ damage in the colonic epithelial counterpart [20]. Here, we describe, for the first time, a specific Th17-dependent gene expression pattern in patients with chronic venous disease compared to controls. The key expression markers of the CD4 + Th17+ switch were strongly activated (IL23R/IL17/ROR-γ/TGF-β), mainly in CVD and more lightly in advanced ulceration. Moreover, in terms of cell signaling, as expected from IL23R over-expression, also *SGK1* and FOXO1 were over-expressed in the cohort of examined patients compared to healthy controls. The expression markers’ study was also corroborated by correlation analysis, thus demonstrating consistency and inter-dependence on the IL23R-SGK1 pathway. Data have been subjected to ROC analysis defining, for IL23R, IL17, FOXO1, SGK1, and RORγ, a clinical value as potential diagnostic markers. On the other hand, correlation studies by multi-logistic regression showed that analyzed markers correlated with the clinical parameters examined both in the patients and controls, providing a contribution, as empirical risk factors, for the development of venous insufficiency and ulcers. Taken together, the data offer a new horizon for the molecular understanding of chronic venous disease in its various stages of development as dependent on conditions of systemic immune dysregulation, attributable, at least in part, to a shift of the immune response towards the Th17 axis.

## 2. Materials and Methods

### 2.1. Study Design and Procedures

This was a cross-sectional clinical study examining 23 consecutive patients with CVI and CVLUs between May and September 2021. The study was approved by the Institutional Review Board of Interuniversity Center of Phlebolymphology (CIFL) and the International Research and Educational Program in Clinical and Experimental Biotechnology (Approval number: ER.ALL.2018.42.A.), and all patients gave written informed consent. The protocol was properly registered at the public trial registry, www.clinicaltrial.gov (accessed on 11 September 2018) (trial identifier NCT05134597). Salt consumption was assessed by administering a self-assessment questionnaire to patients. The questionnaire was adapted from [1]. Inclusion criteria were patients aged >18 years, with conditions of venous insufficiency of the lower extremities (c2–c4) or venous ulcers (c6), according to CEAP classification [21]. Exclusion criteria: diabetic patients, patients with established autoimmune-based disease conditions, and patients on chronic therapy with glucocorticoid drugs or immunological modulators.

### 2.2. Collection of Blood Samples

Blood samples were collected in 3-mL K3 EDTA vacutainer tubes (Cat. No. 368857, Becton Dickinson, Heidelberg, Germany). Peripheral blood mononuclear cells (PBMCs) were isolated via density gradient centrifugation in a Biocoll Separating Solution (Cat. No. L6115, Biochrom GmbH, Berlin, Germany) within 2 h of sample collection. PBMcs were plated in RPMI-1640 (Life Technologies, Waltham, MA, USA) supplemented with a 10% serum and 1% penicillin-streptomycin solution (Aurogene, Rome, Italy) for 12 h in a 37C incubator in a humidified atmosphere of 5% CO_2_ and 95% air. After adhesion of the monocytes and dentritic cells, PBLs (peripheral blood lymphocytes) were derived and subsequently used for the experiments.

### 2.3. RNA Extraction and qRT-PCR

RNA extraction from PBL was performed using the miRNeasy Mini Kit (Qiagen, Valencia, CA, USA), following the manufacturer’s instructions. Total RNA quantification and quality control were carried out using spectrophotometric analysis (NanoDrop 2000, Thermo Scientific, Waltham, MA, USA) and formaldehyde agarose gel electrophoresis. One microgram of total RNA from each sample was subjected to reverse transcription using the High-Capacity RNA-to-cDNA Kit (Applied Biosystems, Foster City, CA, USA), according to the manufacturer’s instructions. One microliter of cDNA was amplified via real-time PCR using Promega SYBR green (Promega, Madison, WI, USA) and 10 pmol of primers, specific for the target the mRNAs IL23R, IL17A, TGFB, RORG, SGK1, FOXO1, and RANBP1, and for the housekeeping HPRT1 (Supplementary Tables S1 and S2). Real-time PCR assays were performed in triplicate in a total volume of 20 μL using a Bio-Rad CFX96 Touch Real-Time PCR Detection System with the following steps: initial denaturation for 3 min at 95 °C, followed by 40 cycles of 10 s at 95 °C and 1 min at 57 °C. The specificity of the PCR products was determined via melting curve analysis. Values were normalized to the housekeeping gene HPRT1, and the fold expression was determined by the 2^−ΔΔCt^ formula, where ΔΔCt corresponds to the difference between the ΔCt value of each target gene in a subject and the average of the ΔCts in the control cohort.

### 2.4. Immunoblotting

Cells derived from two control CVI groups and CVULs were processed, as indicated in the previous report [2], and probed with different antibodies: a rat-monoclonal ROR-gamma antibody (14-6088-82, Thermo Fischer Scientific, Waltham, MA, USA), a rabbit polyclonal IL-23R antibody (# PA5-113004, Thermo Fischer Scientific), a rabbit polyclonal FOXO1 antibody (# PA5-20972 Thermo Fischer Scientific), a rabbit polyclonal GAPDH antibody (sc-47724, Santa Cruz Biotechnology, Santa Cruz, CA, USA a rabbit polyclonal SGK1 antibody (# 07-315 EDM Millipore Corporation, Temecula, CA, USA), an anti-rat antibody (sc-2006, Santa Cruz Biotechonology, Temecula, CA, USA), an anti-rabbit IgG antibody (# 7074, Cell Signaling Techonology, Danvers, MA, USA), and an anti-mouse Ig antibody (# 7076, Cell Signaling Technology, Danvers, MA, USA).

### 2.5. Statistical Analysis

Continuous variables were reported as either mean ± standard deviation (SD) or median and interquartile (IQR) range based on their distribution. Comparisons among the venous disease categories was assessed by a one-way ANOVA or Kruskall–Wallis test. Categorical variables were analyzed using a chi-square test. Multivariable analyses were performed using ordinal logistic regression under a proportional odds model [22] to evaluate the predictors of changing venous disease categories towards higher values. This approach simultaneously models two cumulative logits that correspond to using binary cut points at the venous insufficiency and venous ulcer, written as log{Pr(venous insufficiency)/Pr(control group)} and log{Pr(venous ulcer)/Pr(venous insufficiency or control group)}, respectively. Under this proportional odds model, one coefficient is estimated for each predictor in the model. The coefficient represents the effect of a one-unit increase in the predictor variable on the logit (log odds), which is assumed to be the same for both logits. A score test was used to verify the proportional odds’ assumption in the final model. To compute the risk of venous insufficiency alone, which is considered a first phase of the disease, we built a binary regression analysis by testing the odds ratio for the same covariates as those included in the ordered logistic regression. Both models were adjusted for clinical variables decided “a priori” as potential risk factors for severe venous disease: age, gender, level of salt consumption with diet (low, high, or extreme), and the genetic marker of interest. A two-tailed *p* value of < 0.05 was considered significant for all analyses. Data were analyzed using STATA version 14 (Stata Corp. College Station, TX, USA). Concerning gene expression analysis, differences between groups were analyzed using the nonparametric Kruskal–Wallis test, followed by Dunn’s test for multiple comparisons. All tests were performed at least in triplicate, and all experiments were performed at least 3 times. The Spearman rank coefficient (r) was used to evaluate bivariate correlations. Diagnostic performance of target genes’ expression levels in discriminating across the healthy controls and chronic venous insufficiency (CVI) groups was assessed by areas under the curve (AUC) obtained by the receiver operating characteristic (ROC) by using SPSS software (IBM, Armonk, NY, USA). Gene expression and correlation analyses were conducted using GraphPad Prism software (San Diego, CA, USA, version 5), and differences were considered significant at * *p* ≤ 0.05, ** *p* ≤ 0.01, and *** *p* ≤ 0.001.

## 3. Results

### 3.1. Baseline Characteristics

A total of 23 overall samples, including a control (*n* = 8), the chronic venous disease C2–C4 (*n* = 8), and venous ulcers (C6) (*n* = 7), were included in the study. The patient characteristics are summarized in Table 1 and Table S2. The study population was distributed as follows: the overall male gender was 47.8%, the control of the male gender was 50%, the venous insufficiency of the male gender was 25%, and the venous ulcers of the male gender was 71.4%, with a mean age of 55.9 ± 16.9 years. At the inclusion, the 26% of the overall study participants had hypertension, with a higher prevalence in the venous ulcer group (42.9%), and 4.4% had a history of cardiovascular disease and used antihypertensive drugs (26.1%), statins (13.0%), diuretics (8.7%), antiplatelet agents (30.4%), and anticoagulant agents (4.4%). The exclusion criteria comprised a presence or recent history of neoplastic diseases, autoimmune diseases, diabetes, the use of hypoglycemic drugs, and glucocorticoids for any reason in the month prior to enrollment.

### 3.2. Th17 Gene Expression Evaluation and Protein Expression Validation

**Figure 1:**
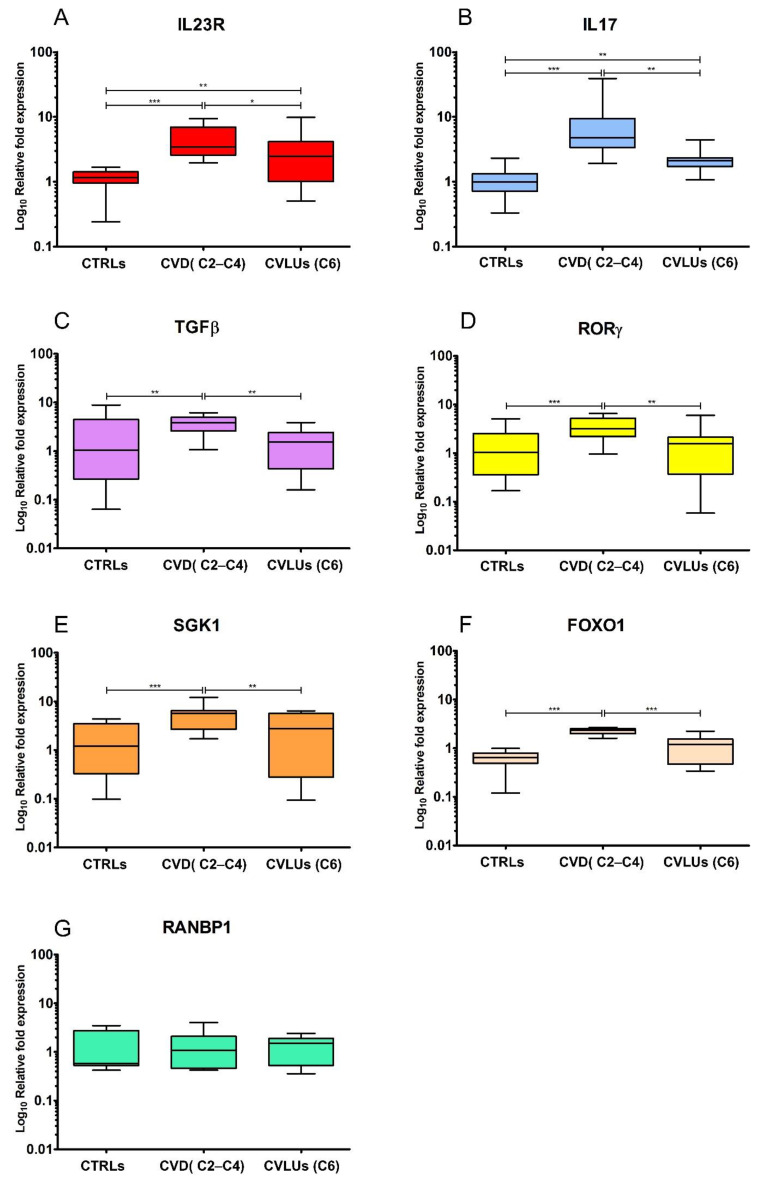
qRT-PCR analysis of IL23R (**A**), IL17A (**B**), TGFB (**C**), RORG (**D**), SGK1 (**E**), FOXO1 (**F**), and RANBP1 (**G**) in PBMCs of the healthy controls, the (*n* = 8), CVD (*n* = 8), and CVLUs (*n* = 7) patients. The results, normalized to HPRT1, are represented as a relative fold expression (Log10 scale) in the whisker plots from minimum (Q0) to maximum (Q4). Statistical significance is indicated at the top of the graph. * *p* ≤ 0.05; ** *p* ≤ 0.01; *** *p* ≤ 0.001.

**Figure 2:**
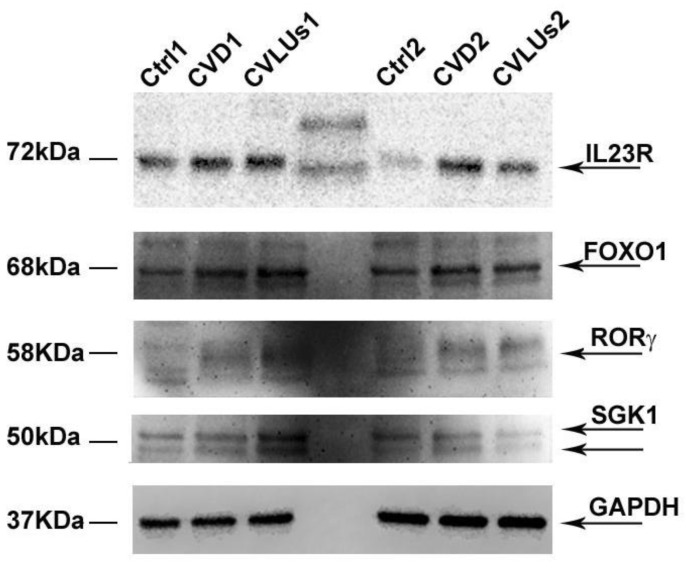
Western blot analysis derived from PBL protein extracts of the controls (No. 2), the CVD patients (No. 2) and CVLUs (No. 2), enrolled in the study. The results normalized for GAPDH show protein expression levels of IL23R, FOXO1, RORg, and SGK1.

To determine whether the Th17 gene expression pattern was involved in the pathogenesis of chronic venous disease, we analyzed in the peripheral blood lymphocytes (PBL) derived from peripheral blood mononuclear cells (PBMCs), the expression levels of the main genes involved in Th17 differentiation: *IL23R*, *IL17*, *RORγ*, *TGFβ*, *SGK1*, *RANBP1*, and *FOXO1* of the healthy controls of the CVD and CVULs’ patients. Surprisingly, all of the gene levels that we examined showed a strong and consistent increase in patients with chronic venous insufficiency compared to the healthy controls and ulcers (Figure 1A–F). On the other hand, *IL23R* and *IL17* also showed a consistent up-regulation in patients with ulcers compared to the controls (Figure 1A,B). No significant change was recorded in the *RANBP1* levels, which, in our previous experiments, varied only in primary Th17 cells and not in PBL (Figure 1G). Taken together, the data show, for the first time, how a strong increase in the expression levels of the main genes involved in Th17 differentiation is present in the early stages of chronic venous disease (CVD) and persists, albeit at a more attenuated level, in the advanced condition of ulceration (CVLUs). In order to determine whether there was a match between the gene and protein expression, some of the markers that we studied were verified for protein expression by immunoblot. Although PBLs in terms of protein expression show a high heterogeneity yet a consistent expression trend, they were sufficiently demonstrated for all of the markers analyzed (Figure 2).

### 3.3. Correlation Study

**Figure 3:**
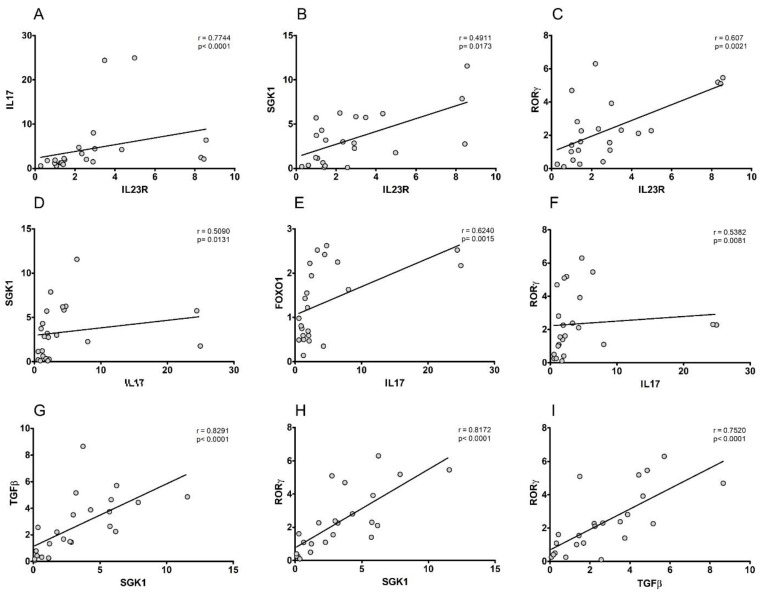
A Spearman correlation analysis on the PBMCs’ gene expression in the overall study subjects (*n* = 23). Data plotted as a fold expression revealed significant positive correlations between IL23R and IL17A (**A**), SGK1 (**B**), RORG (**C**), between IL17A and SGK1 (**D**), FOXO1 (**E**), RORG (**F**), between SGK1 and TGFB (**G**), RORG (**H**), and between TGFB and RORG (**I**). The Spearman rank coefficient (r) and the *p* value are indicated at the top of each graph.

In order to assess the internal consistency and interdependence of the examined markers’ expression levels and to derive information in terms of Th17-signalling, we conducted a correlation study for the observed data, extended to the entire population enrolled in the study. Firstly, our analysis was conducted on the genes whose expression is essential and limiting for defining a successful CD4 + Th17+ differentiation. Interestingly, the data revealed consistent positive correlations between the expression levels of *IL17* and *IL23R* (Figure 3A), *FOXO1* (Figure 3E), *RORγ* (Figure 3F) (r = 0.7744 and *p* < 0.0001, r = 0.6240 and *p* = 0.0015, and r = 0.5382 and *p* = 0.0081, respectively), as well as between *RORγ* and *IL23R* (Figure 3C), *TGFβ* (Figure 3I) (r = 0.607 and *p* = 0.0021, and r = 0.7520 and *p* < 0.0001, respectively). Further, correlation analysis was extended to the *SGK1* expression levels. The data showed consistent correlation in the expression levels of *SGK1* with *IL17* (Figure 3D) and *IL23R* (Figure 3B) (r = 0.5090 and *p* = 0.0131 and r = 0.4911 and *p* = 0.0173, respectively), and a more pronounced positive correlation with *RORγ* (Figure 3H) and TGFβ (Figure 3G) (r = 0.8172 and *p* < 0.0001 and r = 0.8291 and *p* < 0.0001, respectively). Taken together, these findings suggest that, even in CVD, SGK1 may play a critical role in defining a Th17 signature.

### 3.4. ROC and Multivariable Logistic Regression Analysis

**Figure 4:**
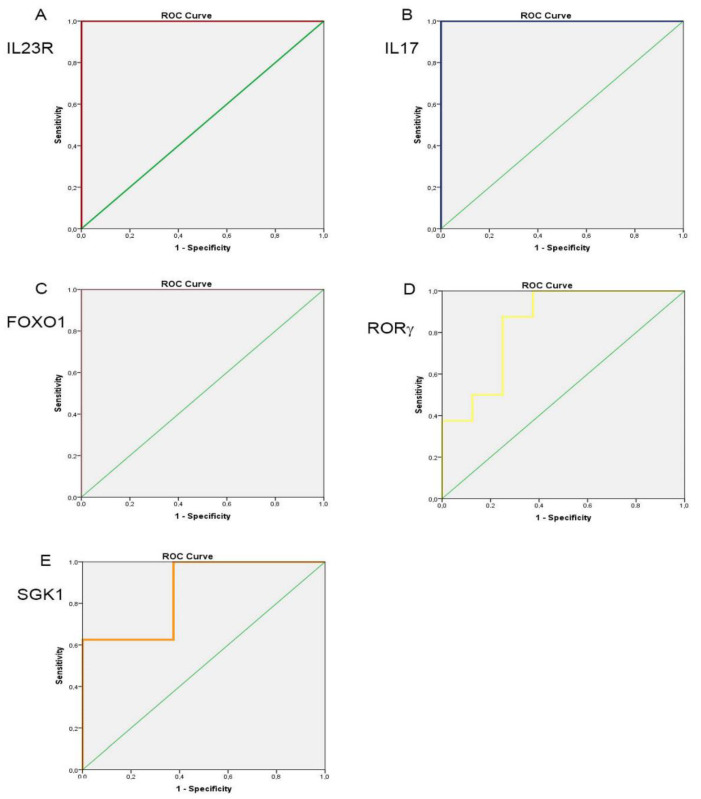
ROC curves for target genes expression levels used to evaluate the discrimination of patients with CVD from healthy controls. AUC of IL23R = 1 (**A**); AUC of IL17A = 1 (**B**); AUC of FOXO1 = 1 (**C**); AUC of RORG = 0.84 (**D**); AUC of SGK1 = 0.86 (**E**). AUC = 0.5 not informative test; 0.5 < AUC ≤ 0.7 not accurate test; 0.7 < AUC ≤ 0.9 moderately accurate test; 0.9 < AUC < 1 highly accurate test; AUC = 1 absolutely informative test.

To estimate the diagnostic performance of the target genes, we performed a ROC analysis to measure the predictive-diagnostic applicability of the expression levels in distinguishing patients with venous insufficiency from the healthy controls. In this regard, an AUC of 1 was calculated for *IL23R* (Figure 4A), *IL17A* (Figure 4B), and *FOXO1* (Figure 4C), with, respectively, a sensitivity of 100% and a specificity of 100% at a cut-off point of the 1.84-fold expression for *IL23R*, a sensitivity of 100% and specificity of 100% at a cut-off point of the 2.20-fold expression for *IL17A*, and a sensitivity of 100% and specificity of 100% at a cut-off point of the 1.30-fold expression for *FOXO1*. On the other hand, an AUC of 0.84 with a sensitivity of 100% and a specificity of 62.5% at a cut-off point of the 1.30- fold expression was computed for *ROR*γ (Figure 4D); an AUC of 0.86 with a sensitivity of 100% and a specificity of 62.5% at a cut-off point of the 1.49-fold expression was computed for *SGK1* (Figure 4E). For *TGFβ*, a lower diagnostic capability based on its expression levels was, instead, observed, as the analysis revealed an AUC of 0.70 with a sensitivity of 100% and a specificity of 62.5% at a cut-off point of the 1.51-fold expression (data not shown). Although a limitation of this analysis is represented by the small sample size of each group, the emerging results are very suggestive and extremely useful in designing future studies with larger sample sizes, aiming to consolidate these genes as biomarkers for the diagnosis/screening of venous insufficiency. To strengthen the putative clinical-diagnostic significance of the obtained data, the gene expression levels of the entire study population were subjected to multi-variable ordered logistic regression analysis by matching them with the clinical-anamnestic data reported in Table 1. In the first analysis, we evaluated the risk of passing from a condition of control to a condition of venous insufficiency and then to a venous ulcer. Four genes out of seven were examined and provided a statistically significant risk index, compared with the clinical parameters analyzed in the study population and providing the following odds ratio with a 95% confidence interval: *IL23R* 1.70 (1.08–2.69) *p* = 0.022; *IL17* 1.12 (1.06–1.30) *p* = 0.041; *RORγ* 1.49 (1.23–2.47) *p* = 0.005, and *RANBP1* 2.27 (1.77–6.70) *p* = 0.016 (Table 2). On the other hand, in the second analysis, we wondered which of the examined genes were most predictive of the risk of going from a healthy to a CVD condition. In this specific case, of the seven examined genes, four provided a statistically significant value of an odds ratio with a 95% confidence interval, respectively: *IL23R* 1.91 (1.04–3.52) *p* = 0.037; *IL17* 5.18 (1.26–11.32) *p* = 0.023; *RORγ* 1.66 (0.95–2.88) *p* = 0.053, and *SGK1* 2.44 (1.05–5.68) *p* = 0.38 (Table 3). Taken together, these data confirm that genes related to the Th17 immune-inflammatory signature might emerge as putative risk factors for chronic venous disease.

## 4. Discussion

CVLU is a severe clinical complication of CVD, and several studies were conducted to understand the mechanisms involved in the CVLU’s onset; however, the evidence still remains elusive. The current literature’s data show that genetic risk factors may play important roles in CVD’s progression [4,23]. Chronic inflammation is a critical event in CVLU’s pathophysiology and likely leads to the activation of leukocyte-dependent pathways in response to several cytokines [24]. Several lines of evidence depict a key role of CD4 + Th17+ differentiation in the pathogenesis of different diseases, characterized by a chronic inflammatory stimulation [16] with complete immune dysregulation, loss of the Treg function, and a high-intensity cytokine stimulation [17]. The Th17 inflammatory dysregulation, originally described in cell-mediated autoimmune pathogenesis, is nowadays considered a putative mechanism to explain drug resistance in cancer and HIV, as well as in ulcerative colitis and obesity [11,12,14,15,25]. The serum and glucocorticoid regulated kinase 1 (SGK1) is a serine/threonine kinase that displays several homologies with AKT1, originally described as a steroid, insulin, and cAMP-regulated kinase [26]. Recently, SGK1 [27,28,29,30,31] has also been demonstrated to be involved in cancer as the main effector of the aberrant deregulation of SRC kinase signaling [32], a major driver of cancer development and resistance to therapy [33,34,35,36,37,38]. In the last years, SGK1 has been described as a key gene in Th17 regulation [17,19,20,27], a mediator of the downstream effects of the IL23-receptor [17]. SGK1 is also responsible for the phosphorylative-dependent inactivation of FOXO1, whose translocation in the cytoplasm triggers RORγ activation, leading to Th17 differentiation [17] and to phenotypic effects dependent on Th17 cytokines [39,40]. Chronic venous disease, ranging from its mildest forms (C1–C2) to the most severe form of CVD (skin changes and CVLus) (C4–C6), represents an articulated inflammatory process [1,3]. Little is still known about the crucial role that the innate immune system may play in the predisposition, onset, and pathogenesis of CVD. The data would seem to confirm an initial role of the Th17 lymphocytes in arterial disease [41], whereas in venous disease, only a phlogistic involvement of IL17 [41] and of some other cytokines have been described. However, no reliable data exist regarding the stratification of gene expression and immune involvement in the various phases of chronic venous disease (CVI and CVULs), nor are there present data about the potential diagnostic and predictive value of this signature in CVD.

In this study, after the careful stratification of the patients, for the first time, we measured the gene expression levels of the main Th17 differentiation markers [17,20]. Our experimental findings show a clear-cut up-regulation of *IL23R*, *IL17*, *TGFβ*, *RORγ*, *FOXO1*, and *SGK1* levels in the cohort of patients with CVD. In our opinion, this may demonstrate how a real immune-remodulation, mediated by an initial inflammation, has driven a general Th17 differentiation that, in turn, is able to support a chronic inflammatory phenotype that could fully or partly explain the pathophysiological alterations in CVD. In the cohort of patients with venous ulcers (C6), these differences, although still present, tend to be attenuated. Indeed, in the advanced stages of venous disease, in addition to Th17 stimulation, different phenomena are superimposed, such as regenerative, coagulative, and fibrotic phenomena, pushing towards a more immune-suppressive response. In the canonical Th17 response, feedback is established between the IL23 receptor, SGK1 kinase (autocrine loop), and TGFβ stimulation, thus leading to the differentiation of CD4+ lymphocytes into CD4 + TH17+ lymphocytes [3]. Indeed, activated SGK1 migrates into the nucleus and promotes FOXO1 phosphorylation, which, in turn, acts as a trans-inhibitor of the transcription factor RoRγ [4]. Once phosphorylated by SGK1, FOXO1 is de-localized into the cytoplasm, allowing the free activity of RoRγ in promoting full Th17 differentiation. This leads to the IL17 production as the terminal step [5]. In other experiments performed in primary Th17 cells, RANBP1 [2], the downstream effector of SGK1 that controls the nuclear-pore complex, also appears to be involved in the differentiative process (the data are not shown). However, in our hands, such activation is not found in PBL but only in isolated Th17 cells. In the attempt to determine a correlation between gene and protein expression, immunoblot analysis was performed on the residual protein portion of selected samples from the study population, thus determining that, although slight, differences in the protein expression of the markers examined are present within the conditions under investigation. The slight differences with respect to the gene expression could be attributed to the heterogeneity of the PBL cell population. However, taken together, even from a protein perspective, the data show a trend in line with the previous data.

To better define the association of the examined markers with the pathological phenotype, we carried out a correlation study. As expected, the markers are classically linked to the Th17 phenotype (e.g., IL23R, IL17, RORγ, and FOXO1) and fully positively correlate each other, thus demonstrating a dependence of events. These appear linearly spread along the population under investigation (controls/CVD/CVLUs). From a cell signaling point of view, interestingly enough, all of the markers converge on SGK1. Indeed, as recently demonstrated, SGK1, downstream of IL23R [19], mediates the terminal effects of lymphocyte differentiation that, through the activation of RORγ, lead to the remodulation of the Th17-dependent gene expression [42]. In order to evaluate the potential diagnostic power of the examined gene, we performed several biostatistical analyses. In the first one, a ROC analysis confirmed that five of the examined markers perfectly fell within the accepted cutoffs for major screening/diagnostic markers. Indeed, in our experimental conditions, IL23R, IL17, and FOXO1 displayed in the CVD cohort compared to the controls, the maximum level of sensitivity and specificity (100%), whereas *SGK1* and *RORγ*, in front of a 100% sensitivity, show lower, although acceptable, levels of specificity. This is explained by the fact that SGK1 and RORγ, which are essential and limiting effectors in the differentiation process, are, at the same time, involved in several regulatory processes and also play key roles in multiple immunological pathways, thus explaining a lower specificity [17,39]. However, overall, this analysis confirms, despite a limited sample, a remarkable diagnostic potential for these markers that will require additional studies to be further validated. Finally, we focused on the potential risk factor that this examined gene signature exhibits to the development of pathological conditions, starting from a healthy condition. In this effort, we performed a multi-logistic regression analysis by pairing the clinical data of the patients and controls examined with the gene expression values. Firstly, we investigated which of the examined markers provided a statistically significant risk factor to move from a healthy condition to CVD and then to ulceration. The evidence, although a net of a limited study population, demonstrate that four of the examined genes (*IL23R*, *IL17*, *RORγ*, and *RANBP1*), if taken as a whole and paired with clinical data, provided an extremely clear estimate of risk. In the second analysis, we instead questioned which gene expression levels provided an estimated odds ratio of moving from a healthy condition to CVD, excluding the ulcerative condition and focusing on that cohort with a maximum Th17 expression. In this case, four of the genes examined, *IL23R*, *IL17*, *SGK1*, and *ROR*γ, always agreed in our analysis. The latter definitely represents the genes of the canonical Th17 differentiation pathway [17]. The overall data offer us a new perspective on chronic venous disease. It is possible to figure out that, as a result of the various chronic stimuli known (including diet, BMI, smoking, cardiovascular disease, and other genetic factors [1]), there may be a systemic rearrangement of the innate immunity, able to induce a selective pressure towards a Th17 differentiation. In turn, Th17 lymphocytes could be responsible for the inflammatory maintenance and vascular remodeling, able to lead to the full development of CVD [43]. The study of Th17-gene markers represents not only a new mechanism of pathophysiological understanding but may help clinicians to identify CVI and CVLUs forms that should require advanced wound-healing treatment modalities, thus developing also new therapeutic approaches with prognostic/diagnostic value.

## 5. Study Limitations

This study, which has the potential to highlight how the Th17 immune regulatory axis may be involved in the pathogenesis and evolution of CVD, currently has the limitation of the small number of the study population. The data, if confirmed in future larger-scale studies, highlight that a risk measure and prognostic assessment of the immune aspect of CVD can be obtained from a limited peripheral blood sample. This enforces the need to continue with this type of investigation, expanding the enrolled population in order to better validate and deepen the significance of the current results.

## Figures and Tables

**Table 1:** Baseline characteristics of overall patients and for the categories of venous disease.

	Overall (N = 23)	Control (N = 8)	CVD (C2–C4) (N = 8)	Venous Ulcer (C6) (N = 7)	*p*
Age, years	55.9 ± 16.9	51.4 ± 16.3	52.5 ± 11.6	65.0 ± 21.1	0.046
Male gender, %	47.8	50.0	25.0	71.4	0.197
Body Mass Index, Kg/m^2^	28.3 ± 6.2	28.4 ± 4.9	27.5 ± 8.0	29.0 ± 6.2	0.905
Smoking habit, %	8.7	12.5	12.5	0	0.619
History of Cardiovascular D. %	4.4	0	0	14.3	0.303
Hypertension, %	26.1	12.5	25.0	42.9	0.408
Systolic BP, mmHg	129.2 ± 12.2	120.0 ± 6.0	130.3 ± 11.1	138.6 ± 11.1	**0.006**
Diastolic BP, mmHg	81.8 ± 6.8	81.9 ± 7.0	83.4 ± 5.2	80.0 ± 8.9	0.655
Salt consumption					0.409
Low	17.4	37.5	12.5	0	
High	21.7	12.5	25.0	28.6	
Extreme	60.9	50.0	62.5	71.4	
Antihypertensive drugs, %	26.1	12.5	25.0	42.9	0.408
Statins, %	13.0	12.5	12.5	14.3	0.993
Diuretics, %	8.7	0	12.5	14.3	0.553
Antiplatelet agents, %	30.4	25.0	25.0	42.9	0.693
Anticoagulant agents, %	4.4	0	12.5	0	0.375

Cardiovascular D., cardiovascular disease; BP, blood pressure.

**Table 2:** Multivariable ordered logistic regression analyses for the predictors of change status from CTRL to CVD and to CVULs.

Characteristics	OR (95% CI)	*p*
**IL23R**	**1.70 (1.08–2.69)**	**0.022**
**IL17**	**1.12 (1.06–1.30)**	**0.041**
**RORG**	**1.49 (1.23–2.47)**	**0.005**
**RANBP1**	**2.27 (1.77–6.70)**	**0.016**
SGK1	1.17 (0.88–1.56)	0.292
TGF-β	0.87 (0.57–1.31)	0.492
FOXO1	2.58 (0.82–8.06)	0.104

OR, Odds Ratio; CI, Confidence Interval. All ordered logistic models are adjusted for age, gender, and daily salt consumption (low, high, or extreme).

**Table 3:** Multivariable logistic regression analyses for the predictors of CVD.

Characteristics	OR (95% CI)	*p*
**IL23R**	**1.91 (1.04–3.52)**	**0.037**
**IL17**	**5.18 (1.26–11.32)**	**0.023**
**RORG**	**1.66 (0.95–2.88)**	**0.053**
RANBP1	1.31 (0.38–4.45)	0.667
**SGK1**	**2.44 (1.05–5.68)**	**0.038**
TGF-β	1.78 (0.97–3.25)	0.062
FOXO1	2.24 (0.65–5.12)	0.231

OR, Odds Ratio; CI, Confidence Interval. All logistic models are adjusted for age, gender, and daily salt consumption (low, high, or extreme).

## Data Availability

Not applicable.

## Supplementary Materials

The following supporting information can be downloaded at: https://www.mdpi.com/article/10.3390/biom12070902/s1, Table S1: primer sequences; Table S2: Lipidemic status of the study population.
