# Supplementary material for: Th17-Gene Expression Profile in Patients with Chronic Venous Disease and Venous Ulcers: Genetic Modulations and Preliminary Clinical Evidence
Source: Biomolecules. 2022 Jun 28;12(7):902. doi: 10.3390/biom12070902 (PMC9312858; doi:10.3390/biom12070902)
Supplement: Supplementary file 1 [file biomolecules-12-00902-s001.zip › biomolecules-1764972-supplementary.pdf]

Table S1. primer sequences.

mRNA	Sense primer (5'→3')	Antisense primer (5'→3')	Tm primers (Sense/Antisense) °C	Amplicon size	Reference
hsa-IL23R	TACTGGCAGCCTTGGAGTTCA	TAAGGTGCCCTGTAGAGATGGA	57.8/56.3	133 bp	
hsa-IL17A	TACAACCGATCCACCTCACCTT	ACTTTGCCTCCCAGATCACAGA	61.09/61.36	82 bp	(Spagnuolo et al.)
hsa-RORC	GCAGCGCTCCAACATCTTCT	ACGTACTGAATGGCCTCGGT	57.7/57.9	111 bp	
hsa-RANBP1	ATGCGGGCAAAACTGTTCCGAT	ATGGCCCCTTTCTCCTTGTGCT	60.0/60.5	107 bp	(Dattilo et al.)
hsa-SGK1	GGCACCCTCACTTACTCCAG	GGCAATCTTCTGAATAAAGTCGTT	59.7/57.8	102 bp	(Dattilo et al.)
hsa-TGFB1	AATTCCTGGCGATACCTCAGCA	AAGGCGAAAGCCCTCAATTTCC	58.1/58.1	129 bp	
hsa-FOXO1	TCACGCTGTCGCAGATCTAC	GAACTTGCTGTGTAGGGACAGA	56.6/56.3	128 bp	
hsa-HPRT	TGACACTGGCAAAACAATGCA	GGTCCTTTTCACCAGCAAGCT	59.5/61.1	94 bp	(Vandesompele et al.)

Table S2: Lipidemic status of the study population

Controls	Hyperlipidemia [Yes/no]	Triglycerides [30-200mg/dl]	HDL [35-65mg/dl]	Total cholesterol [30-200mg/dl]
1	yes	241	46	200
2	no	85	65	180
3	no	67	53	166
4	no	48	64	154
5	no	65	58	128

6	no	72	45	122
7	no	101	63	168
8	no	67	52	136

CVD	Hyperlipidemia [Yes/no]	Triglycerides [30-200mg/dl]	HDL [35-65mg/dl]	Total cholesterol [30-200mg/dl]
1	no	46	38	159
2	no	87	66	135
3	no	92	39	151
4	no	56	45	175
5	no	62	42	165
6	no	68	62	154
7	yes	104	30	253
8	no	69	58	153

CVLUs	Hyperlipidemia [Yes/no]	Triglycerides [30-200mg/dl]	HDL [35-65mg/dl]	Total cholesterol [30-200mg/dl]

1	yes	232	32	176
2	no	43	39	135
3	no	103	61	164
4	no	65	37	132
5	no	44	67	144
6	no	58	43	161
7	no	47	60	133
